# Lysophosphatidic acid (LPA) as a pro-fibrotic and pro-oncogenic factor: a pivotal target to improve the radiotherapy therapeutic index

**DOI:** 10.18632/oncotarget.16672

**Published:** 2017-03-29

**Authors:** Chloé Rancoule, Sophie Espenel, Jane-Chloé Trone, Julien Langrand-Escure, Alexis Vallard, Amel Rehailia-Blanchard, Anis El Meddeb Hamrouni, Yaxiong Xia, Jean-Baptiste Guy, Majed Ben-Mrad, Nicolas Magné

**Affiliations:** ^1^ Radiotherapy Department, Lucien Neuwirth Cancer Institute, Saint-Priest-en-Jarez, France; ^2^ Cellular and Molecular Radiobiology Laboratory, CNRS UMR 5822, IPNL, Villeurbanne, France

**Keywords:** cancer, radiation therapy, fibrosis, lysophosphatidic acid, proliferation

## Abstract

Radiation-induced fibrosis is widely considered as a common but forsaken phenomenon that can lead to clinical sequela and possibly vital impairments. Lysophosphatidic acid is a bioactive lipid involved in fibrosis and probably in radiation-induced fibrosis as suggested in recent studies. Lysophosphatidic acid is also a well-described pro-oncogenic factor, involved in carcinogenesis processes (proliferation, survival, angiogenesis, invasion, migration). The present review highlights and summarizes the links between lysophosphatidic acid and radiation-induced fibrosis, lysophosphatidic acid and radioresistance, and proposes lysophosphatidic acid as a potential central actor of the radiotherapy therapeutic index. Besides, we hypothesize that following radiotherapy, the newly formed tumour micro-environment, with increased extracellular matrix and increased lysophosphatidic acid levels, is a favourable ground to metastasis development. Lysophosphatidic acid could therefore be an exciting therapeutic target, minimizing radio-toxicities and radio-resistance effects.

## INTRODUCTION

Radiation therapy (RT) is a major anti-cancer local treatment. However, RT induces damages in both tumour cells and healthy tissue located in the treatment fields. Radiation-induced fibrosis (RIF) is one of the most impacting late toxicities, sometimes associated with life-threatening consequences [1]. Fibrosis consists in an accumulation of extracellular matrix leading to a loss of normal tissue architecture, and possibly to organ dysfunction [2]. The lysophosphatidic acid (LPA) is a lipid acting at the intersection of fibrosis and cancer development. LPA's pro-oncogenic (pro-proliferative, pro-angiogenic, anti-apoptotic, and pro-migration / invasion [3–5] properties have been recently evidenced, indeed. Increased secretion of LPA can be triggered by tissue damage (including surgery, chemotherapy and radiation therapy) and/or by chronic inflammation. In these situations, LAP was also shown to have a pro-fibrotic role in various organs [6] [7]. LPA could therefore be a promising therapeutic target to improve the RT therapeutic index (efficacy/toxicity ratio), with the limitation of radio-induced injuries and the optimization of the radio-induced death of tumour cell. The present review summarizes the implications of LPA in the RIF and in the pro-tumour signalling pathways, and proposes it as a key modulator of the radiotherapy therapeutic index. Finally, radiation-induced transformations in the tumour micro-environment will be described, as they probably also promote metastases development.

## RADIATION-INDUCED FIBROSIS

The inflammatory response induced by RT is followed by cell signalling cascades leading to the development of a RIF [8]. Monocytes are recruited and differentiate into macrophages due to an increased secretion of inflammatory chemokines and cytokines. Macrophages secrete platelet-derived growth factor (PDGF, inducing neo-angiogenesis [9]) and TGF-β (inducing differentiation of fibroblasts into myofibroblasts [10]). Neoformed myofibroblasts overproduce the components of the extracellular matrix (collagens, fibronectin) [11], creating a non-elastic tissue. Fibrosis also impacts the blood vessels, with thrombosis probably hindering normal cicatrisation processes [12], leading to an a vicious circle. Risk factors of RIF have been identified: radiotherapy characteristics (dose, fractionation, irradiated volume) [13], other anti-cancer treatments such as chemotherapy [14] or surgery [15], genetic anomalies [16] or tissue diseases [17]. If the first biological phenomena of fibrogenisis appear in the few hours or days following irradiation [1], the “clinical” fibrosis usually appears several months after RT and is mostly considered as irreversible [11]. Of course, the impact for the patient depends on of the primary tumour location, defining the dose, fractionation, the radiation nature and the localization of the RT fields [18]. In head and neck cancer treatment, RT can ultimately cause osteoradionecrosis, xerostomia, dysphagia, dysphonia, trismus, cutaneous fibrosis, plexopathy [19]. After a thoracic RT, the commonly reported late adverse events are lung fibrosis, oesophageal complications (dysphagia, haemorrhages), and cardiac toxicities (including pericarditis and coronaropathy). For abdominal and pelvic tumour locations, dysuria, dyspareunia, loss of fertility, urinary or rectal incontinence are the most feared complications. Therefore, common radio-toxicities may dramatically impact the patient's quality of life and even sometimes be life-threatening [1]. The management of fibrosis mainly includes common anti-inflammatory treatments, antioxidants or vascular therapies, with limited levels of evidence and uncertain efficacy [20]. New challenges have emerged with the comprehension of RIFs pathogenesis. Many signaling pathways have been described, each representing a potential therapeutic target able to reduce the matrix accumulation and the auto-maintained inflammation [12] [21]. If some of these targets appeared promising with strong pre-clinical rationales (as TGF-β, VEGF and certain integrin receptors [1,22]), none really proved efficacy when tested in humans. Recently, another target was identified: the lysophosphatidic acid (LPA), involved in post-RT pro-fibrosis biological processes [7] [23], but also in pro-tumour signalling pathways [3] [4].

## LPA AND FIBROSIS

In the last decades, lipids were identified as mediators able to induce different biological activities, acting as second messengers or as a paracrine/endocrine factors. Cell membrane lipid derivatives (or lysophospholipid (LPs)) were shown to proceed as extracellular signals, joining other lipidic mediators including prostaglandins, leukotriens, platelet-activating factors or endocannabinoids [24]. The lysophosphatidic acid (LPA) is the simplest natural glycerophospholipid. It can be considered ubiquitous, as it was identified in many tissues [25] and biological fluids [26]. The extracellular LPA is though to be mainly produced by the autotaxin (ATX), a lysophospholipase D transforming LPs in LPA [27, 28]. LPA acts through 7 specific trans-membrane domains coupled to G proteins receptors (LPARs). If the exact role of LPA, LPARs, and ATX among tissue remains to be clearly determined, it is hypothesized to be of primary importance since knock-outing ATX [29] [30] [31] is generally lethal, with important blood vessels and neurologic malformations. LPA is considered as a growth factor-like phospholipid, inducing proliferative and/or morphological effects in almost every cell type [32], but recent studies also indicated that LPA significantly participated in the normal wound healing process (cicatrisation) and in the degenerated wound healing process (fibrosis) [26]. The exact LPA secretion process (which cells are responsible for production, the mechanisms of tissue injury related to specific LPA signalling pathways) in the case of fibrosis is still poorly understood [33]. Cells partly “responsible” (but they do not produce it, neither stock it) for the plasmatic LPA secretion could be platelets, as circulating LPA was shown *in vitro* to results of the enzymatic hydrolysis by ATX [34] of platelet derived phospholipids [35]. However, there is no *in vivo* evidence for LPA or ATX secretion by any cell type, even if it is hypothesised that lymphatic high endothelial venules and adipose tissue could be possible ATX sources [33]. LPA and ATX are reported to be produced in response to inflammation to mediate wound repair [36], probably through a tumour necrosis factor (TNF) enhanced secretion. TNF, a major pro-inflammatory cytokine, was indeed shown to increase ATX expression from fibroblasts [37] and hepatoma cell lines [38], through NF-κB over-expression [38]. If the inflammation is not resolved, high ATX and LPA concentrations persist, leading to inflammatory or fibrotic diseases, and possibly to cancer [36, 39, 40]. In various animal models of kidney, lung, and dermal fibrosis, authors reported an increased LPA production and an increased expression of LPARs (LPA1R, LPA3R) [26] [41]. Increased LPA and LPARs expressions have also been observed *in vitro* in arterial [42] and in liver fibrosis in human and animal models [43–45]. If data associating LPA with fibrosis were interesting evidences of the LPA's potential impact on tissue repair dysfunction, the fact that the inhibition of it signalling prevented from fibrosis was of primary importance, proving that this pathway could be a valuable therapeutic target. The first step was the development of LPA1R-deficient mice after pre-cited studies reported increased LPA levels (in murine and human assessments) in case of bleomycine-induced lung fibrosis. Two weeks after toxic exposure to bleomycine, lung collagene, peribronchiolar and parenchymal fibrosis were significantly decreased in LPA1R-deficient mice *versus* wild type ones [46]. These observations were corroborated by the fact that lung fibroblast recruitment and vascular leak (two major phenomenon leading to lung fibrosis [47]) were significantly decreased in LPA1R-deficient mice. Human fibroblasts were then tested, and shown to be more attracted when the bronchoalveolar fluid was rich in LPA (*ie* obtained from patients with a lung fibrotic disease). Interestingly, this effect could be totally reversed when applying a LPA1R inhibitor (Kil6425) [46]. These data were corroborated by other studies inhibiting LPA or its signalling pathways (ATX or LPARs). The AM095, a LPA1R selective antagonist, was tested *in vivo* on murine models of lung and kidney fibrosis [48]. After bleomycine-induced lung damages, AM095 significantly decreased collagen, protein and inflammatory (macrophage and lymphocyte) cell infiltration detected in bronchoalveolar fluid. After hypoxia-induced kidney damages, AM095 decreased renal fibrosis in treated mice [48]. Other LPARs inhibitors have successfully reduced fibrosis induced by hypoxia or bleomycine in murine models [41] [49], making LPARs some potential interesting therapeutic targets. To our best knowledge, at least three LPA1R antagonists are currently tested in phase I/II clinical trials for idiopathic pulmonary fibrosis or systemic sclerosis [50] [51]. LPA was therefore recently studied in the specific context of cancer [52], with a special focus on radiation-induced injuries. Deng *et al.* suggested that interaction between RIF and LPA/LPAR could be more complex than in fibrosis induced by other causes [23]. In murine models exposed to radiation, the presence of LPAR2 was necessarily to protect animals from radiation induced intestinal injury, indeed. The octadecenyl thiophosphate (OTP), a complete antagonist of LPA2R, was tested as a radioprotective drug, and showed that it could decrease the radiation-induced apoptosis. OTP, when delivered intraperitoneally, reduced death caused by lethal dose 100/30 radiation by 50%, but had no effect in LPAR2 knockout mice [23]. The role of LPA receptors 1 and 3 has also been explored. In murine models, the blockade of LPAR1/3 with a receptors antagonist ameliorated radiation pneumonitis and radiation-induced lung fibrosis [7] [53]. However, few data are available and further pre-clinical studies are expected in the specific field of RIF.

## CANCER AND LPA

**Figure 1:**
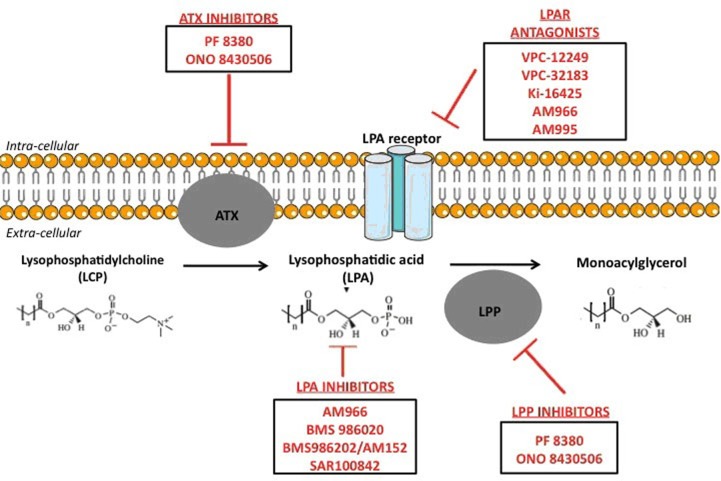
Kgkfhdhdfhfdhfdhfd hdfhfdh Experimental treatments targeting LPA pathways.

In parallel of its pro-fibrotic activity, LPA has also been repeatedly described as an oncogenic promoter, responsible of tumour initiation, growth, and metastasis [3–5, 54, 55]. In different primary tumours (*e.g*. glioma, small cell lung, renal, liver, breast, ovarian and thyroid cancers [3, 4, 54]), as well as in cancer patient plasma [55], increased ATX expression and therefore increased LPA levels were reported, suggesting LPA as a potential cancer biomarker [56]. Besides, LPA was shown to stimulate important oncogenic signalling pathways. For instance, it was reported that LPA could stimulate production of VEGF [57], interleukine-8 (IL-8) [58] and urokinase plasminogen activator (uPA) [59] in ovarian cancer cells, enhancing hyper-vascularisation processes [3] finally leading to metastases. Furthermore, it was shown *in vitro* that LPARs (proteins transducing the LPA signal) controlled crucial cellular functions. LPAR1-4 stimulate the mitogenic pathway Ras-Ras-Raf-MEK-ERK pathway, and the pro-survival PI3K pathway [60]. LPAR1-3 and LPAR5 stimulate the invasion/migration thought the activation of the Rho pathway. The Rac Pathway, also involved in invasion/migration, can be stimulated by LPAR1-4 as well [60]. The direct role of LPA3R in invasion/migration processes was proved in a recent publication of Okabe *et al* [61]. LPAR3-expressing cells were created from murine hepatoma cells and compared with LPAR3-unexpressing cells, regarding their migration and tumorigenicity abilities. LPA significantly increased motility and invasion of the LPAR3-expressing cells, compared to LPAR3-non-expressing cells. Interestingly, this phenomenon could be inhibited with inhibitors of Gi or Gq protein (*ie* proteins resulting of LPARs activation by LPA, stimulating RaS-, Rac-, Rho- and PI3K- signalling pathways [60]) [61]. Authors even demonstrated *in vitro* that LPA3R expression was a factor of resistance to cisplatin and docetaxel [61]. The direct implication of LPA in invasion/migration processes was also demonstrated *in vitro* and *in vivo* in murine models [62]. *In vitro*, LPA significantly increased ovarian cancer cell invasion, partly through the down-regulation of invasion negative regulators (tissue inhibitor of metalloproteinases (TIMPs)) [62]. Interestingly, authors clearly showed that LPA specifically induced invasion in primary and metastatic malignant tumour cells, and not at all in normal cells. *In vivo*, LPA stimulated ovarian tumour metastasis, but this phenomenon could be significantly inhibited using an inhibitor of PI3K (an effector in the LPA signalling pathway) [62]. It was also recently shown that the epithelial to mesenchymal transition (EMT) (required for the invasion/migration process) was increased by LPA, in ovarian cancer cells lines [63]. When treated with LPA, ovarian cancer cells multiplied by 150 the HIF1α secretion *in vitro*, through the Gαi2 protein secretion (a protein activated by the LPAR). As HIF1α is a well-known promoter of EMT, authors tested if LPA stimulated both EMT and invasion/migration. The *in vitro* assessment clearly stated that it did, through increased secretions of N-cadherin and Slug/Snail2. These data were corroborated *in* vivo by Kim *et al.,* who showed in a murine model that injections of LPA stimulated the ovarian cancer metastasis development, with increased size and variety of locations [64]. A clinically validated HIF1α-inhibitor (PX-478) was very recently tested *in vitro* and successfully inhibited the invasion/migration process of ovarian cancer cells [63]. Of course, such results with LPA/LPAR inhibitors seem promising and the further *in vivo* pre-clinical or clinical results are eagerly awaited. LPA/LPAR inhibitors are summarized in Figure 1.

Another very interesting action of LPA and LPARs is the negative regulation of the immune system, and especially of the cytotoxic CD8 T lymphocytes that normally detect and eliminate nascent tumours. As it was recently proved that several LPARs were expressed by T lymphocytes [60] [65], their role in the cancer immune response was explored. LPA5R (when activated by LPA) was specifically identified as an inhibitor of the CD8 T lymphocytes antigen receptor [66]. LPA (through LPA5R) inhibited *in vitro* and *in vivo* the CD8 T lymphocytes antigen receptor signalling*,* and the subsequent cytotoxic T cell activation and proliferation [66]. Authors showed in a mouse melanoma model that animals receiving LPAR5-deficient tumour-specific CD8 T lymphocytes controlled tumour growth significantly better than animals receiving wild type CD8 T cells [66]. These results suggest that LPA5R blockade could be an efficient strategy to promote host CD8 T cell anti-tumour immunity.

Furthermore, it was suggested that hypoxia (*i.e.* the environment where are located the most aggressive and radio-resistant cancer cells [67]) could additionally favour LPA activity. *In vitro*, ovarian tumour cells’ invasion/migration due to LPA was enhanced if cells were placed in hypoxic conditions, probably due to an increased HIF1α secretion [64]. These data were corroborated by the observation of ATX-deficient animals having collapsed HIF1α secretion. It also seems that LPA stimulates HIF1α secretion *in vitro* [68] and *in vivo* [6], creating an auto-maintained circle of amplification. The links between LPA and hypoxia are of primary importance considering that hypoxia is an independent factor of tumour radiation-resistance, as oxygen enhances the creation of radio-induced free radicals that ultimately damage DNA and induce clonogenic cell death. Finally, the ATX-LPA axis is up-regulated in tumours due to 3 concomitants phenomenon: An increased ATX secretion leading to high LPA levels, an increased LPARs expression on tumour cell surface, and a down-regulation expression of molecules degrading LPA [36] [39]. This triad creates a vicious circle, stimulating all the pre-cited pro-proliferation, -migration, -metastasis and -therapy resistance pathways.

## LPA AND RADIATION RESISTANCE

**Figure 2:**
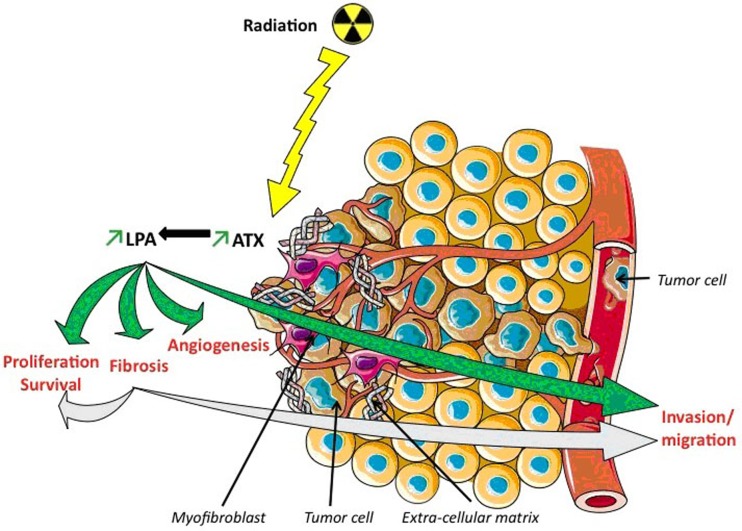
Pro-fibrotic and pro-oncogenic effects of lysophosphatidic acid (LPA) induced by radiation therapy In Green: The evidenced-based activation of LPA, increased by radiations, through ATX. Ionizing radiations increase AUTOTAXIN (ATX) gene expression and lysophosphatidic acid (LPA) levels. LPA participates to the enhancement of fibrosis, inducing recruitment of myofibroblasts responsible of extra-cellular matrix accumulation. LPA participates to radioresistance phenomenon inducing angiogenesis, proliferation, survival and invasion/migration of tumor cells (green arrows). In Blue: Our hypothesis: In addition to the pre-cited phenomena, the mediators of fibrosis promote cancer local and distant development. Fibrosis pathogenesis and extra cellular matrix components could also lead to radioresistance through similar mechanisms (light blue arrows).

Regarding the interaction between LPA and radiotherapy, data are still scarce. However, two biological concepts are supported by solid observations: LPA secretion induces tumour radiation-resistance and radiotherapy induces LPA secretion Indeed, in animal models, local and systemic levels of LPA were reported to significantly rise after exposure to radiation [69,70], doubling in case of total body irradiation [71]. To our knowledge, no assessments have been performed in daily routine radiotherapy, and our team is currently recruiting patients in order to monitor LPA levels before, during and after radiotherapy courses. Furthermore, radio-resistance studies were conducted and revealed that LPA decreased radiation-induced cell death. For instance, Deng et al. have shown that LPA reduced apoptosis of nontransformed intestinal crypt-derived epithelial cell line, when administered 1 h before or 2 h after a high dose radiotherapy (>10Gy) [72]. Interestingly, they also showed that LPA not only decreased the number of radiation-induced apoptotic cells, but also rescued apoptotically condemned cells, indicating that LPA had a radioprotective effect. The same team showed later that the anti-apoptotic effect of LPA was due to the specific activation of LPA2R [23], inhibiting the mitochondrial apoptosis cascade [73]. The phenomena involving LPAR2 after radiation are probably complex since both the administration of LPA or OTP (a full antagonistic of LPAR2) in mice undergoing high dose radiotherapy (6-12 Gy) decreased radiation-induced mortality in all animals, but in LPA2R-deficient ones [23]. The crucial role of LPA2R was also proved in vitro, with recent results showing that radiotherapy was more likely to induce apoptosis in LPA2R-deficient cells, and that cells with a LPA2R-knock in were more radiation-resistant, with a ligand-dependent manner [74]. Therefore, LPA2R seem to have unique role in radiation resistance, unlikely to other LPARs. This hypothesis was supported by Lin *et al*., who showed the specific capacity of LPA2R (and not of other LPARs) to bind zinc finger proteins, and especially Siva-1 [75]. Siva-1 is a protein of major importance, as it is produced after DNA-damage and makes the mitochondrial outer membrane more prone to apoptosis [76]. Once Siva-1 and LPAR2 are bound, the complex is degraded. Therefore, the LPAR2 activation is thought to result in the depletion of the cell for Siva-1, leading to the attenuation of apoptotic signalling. Moreover, it was shown that LPA2R had the ability (when activated by LPA, and unlikely to other LPARs) to bind the thyroid hormone receptor interacting protein 6 (TRIP6). TRIP6 has also a binding motif that can interact with NHERF2, forming a LPAR2–TRIP6–NHERF2 complex that was shown to up-regulate prosurvival pathways such ERK1/2, PI3K-Akt and NF-κB, leading to increased DNA repair and [69] decreased post-radiotherapy apoptosis [77]. Therefore LPA2R, over-expressed in many aggressive tumours, is considered as an interesting therapeutic target. Another anti-apoptotic effect of LPA is also suggested, with a ceramide pathway deregulation [39]. Radiation induced cell death is indeed though to be mediated by a pro-apoptotic sphingolipid, named ceramide [78], that activates caspase and ultimately leads to release the mitochondrial cytochrome C [79, 80]. In cancer cells, the intra-cellular elevation of ceramide following a radiotherapy course is though to partly cause of their death [81–83]. It was recently proved in cancer cells that LPA could decrease the intra cellular ceramide formation [84–86] and increase sphingosine 1-phosphate (S1P, a sphingolipid analogue, counterbalancing ceramide action in the survival *vs* death balance [84–87]). Therefore, an early inhibition of LPA/LPAR could be efficient, for instance using ATX inhibitors [88]. The PF-8380, an ATX inhibitor was tested *in vitro* and *in vivo* and showed a radio-sensitizing effect in murine glioma and humain glioblastoma cell lines [89]. After a 4 Gy irradiation, a decreased clonogenic survival, migration and invasion in cells treated with the ATX inhibitor was assessed. Interestingly, authors showed that the PF-8380 inhibited the radio-induced secretion of Akt (a central downstream target of the RTK/RAS/PI3K pathway, implicated in angiogenesis, cell survival, proliferation, and migration). *In vivo*, in an heterotopic mouse models of glioblastoma, tumour growth was delayed by ≥20 days in animals receiving radiotherapy plus PF-8380 *versus* exclusive radiotherapy [89]. Thus, there is a real pre-clinical rationale that leads to hypothesize that LPA acts as a double-edged sword: it promotes cancer development at local and systemic level, and reduces the effectiveness of radiation therapy. Worst, as LPA induces fibrosis, it probably exacerbates another pro-tumour mechanism. Biological hypothesises regarding the pre-cited phenomena are depicted in Figure 2.

## FIBROSIS AND CANCER

The microenvironment of a fibrotic tissue could play a key role on cancer cells. Indeed, mutual proliferative and/or morphological pathways have been described for oncogenic and pro-fibrotic pathways, suggesting that pathogenicity of fibrosis and cancer are intertwined [90]. A recent study of Liu *et al* described the possible links between carcinoma and fibrosis pathways [91]. These results are supported by the fact that tumour cells’ microenvironment, and particularly the extra-cellular matrix (ECM) holds growth factors and death-suppressive signals [92, 93]. The disruption (created by the tumour) of ECM homeostasis is even thought to provide important pro-tumour drivers [94]. For example, it has been described that lung fibrosis increased lung cancer incidence [95]. In another study, breast benign fibrotic phenomena were associated with a predisposition to breast cancer [96]. Furthermore, gene alterations and proteins expression in the ECM of primary tumours could be related to the overall and progression-free survival [97–102]. These data highlight the critical role of ECM on tumour development, with the regulation of tumour growth through paracrine interactions between tumour and the ECM [103], involving angiogenesis [104]. Fibrosis and cancer seem therefore inextricably linked but more investigational researches are needed to really understand this interaction. The common pathways between fibrosis and carcinogenesis are briefly summarized in the present section [105, 106].

### TGFβ pathway

TGFβ pathway is a well-known fibrosis promoter [107], especially secreted in case of post-radiotherapy lung fibrosis [108]. However, TGFβ is also known to induce a pro-oncogenic activity in advanced cancers [109], with emerging evidences of a Pi3K pathway co-activation [110].

### Connective Tissue Growth Factor (CTGF)

CTGF is a pro-fibrotic factor, mainly known for its role in vascular fibrosis [111]. In cancer, CTGF deregulation (*ie* overexpressed compared to low-expressing normal tissue or underexpressed compared to high-expressing normal tissue), was related to local and distant cancer progression, promoting proliferation, drug resistance, angiogenesis, adhesion, invasion and migration [112].

### PDGF

PDGFs are pro-fibrosis factors, described in kidney, lung, liver, and skin fibrotic diseases [113], as well as in post-radiotherapy lung fibrosis [108]. PDGF receptors were found over expressed in prostate cancer, and glioblastoma cells. Besides, PDGF was found to be excessively activated in brain areas housing glioblastoma cancer stem cells [114]. Strong pre-clinical rationales showing that PDGF-D inhibition reduced tumour angiogenesis or metastases development in renal and pancreatic carcinomas have recently been reviewed [114].

### Wnt

Abnormal activation of Wnt signaling pathway was described in many pathological fibrotic processes [115]. In parallel, cancer stem cell development was reported to be supported by Wnt pathway over-activation [116].

### Notch

Notch pathway was recently shown to stimulate fibrogenesis [117] and promote melanoma progression [118].

We therefore can hypothesize a new concept relating radiotherapy, fibrosis and cancer: radiotherapy induces tissue damages, and therefore stimulates ATX and LPA secretions. LPA promotes fibrosis and tumour radiation resistance. Moreover, the newly formed ECM participates in the cancer development and metastases enhancement, leading to a decreased therapeutic index of radiotherapy (Figure 2).

## CONCLUSION

LPA could be an exciting therapeutic target, optimally minimizing radio-toxicities and radio-resistance effects and improving the therapeutic index. A large arsenal of pharmacological therapies targeting the LPA signalling has been deployed: inhibitors of the autotaxin activity, antagonists of one or several LPAR or monoclonal antibodies against LPA (Figure 1) Pre-clinical and early clinical trials should soon investigate the LPA pathway blockade and its impact as a radio sensitizer but also as protector of healthy tissue. If such effects are confirmed, a pharmaco-bio-modulator agent of tumour's treatment sensitivity and healthy tissues’ treatment protection would be identified for the first time. LPA modulating agents might challenge the modern radiobiological concepts, minimizing oncogenic and pro-metastatic factor, improving tumor radio-sensitization and protecting healthy tissue.
